# Motor neuron-derived induced pluripotent stem cells as a drug screening platform for amyotrophic lateral sclerosis

**DOI:** 10.3389/fcell.2022.962881

**Published:** 2022-08-24

**Authors:** Mariana A. Amorós, Esther S. Choi, Axel R. Cofré, Nikolay V. Dokholyan, Marcelo Duzzioni

**Affiliations:** ^1^ Laboratory of Pharmacological Innovation, Institute of Biological Sciences and Health, Federal University of Alagoas, Maceió, Alagoas, Brazil; ^2^ Department of Pharmacology, Penn State College of Medicine, Hershey, PA, United States; ^3^ Department of Biochemistry and Molecular Biology, Penn State College of Medicine, Hershey, PA, United States

**Keywords:** amyotrophic lateral sclerosis (ALS), induced pluripotent stem cells (iPSCs), drug screening, *in vitro* disease model, 3D cell culture, bioinformactics, motor neuron differentiation

## Abstract

The development of cell culture models that recapitulate the etiology and features of nervous system diseases is central to the discovery of new drugs and their translation onto therapies. Neuronal tissues are inaccessible due to skeletal constraints and the invasiveness of the procedure to obtain them. Thus, the emergence of induced pluripotent stem cell (iPSC) technology offers the opportunity to model different neuronal pathologies. Our focus centers on iPSCs derived from amyotrophic lateral sclerosis (ALS) patients, whose pathology remains in urgent need of new drugs and treatment. In this sense, we aim to revise the process to obtain motor neurons derived iPSCs (iPSC-MNs) from patients with ALS as a drug screening model, review current 3D-models and offer a perspective on bioinformatics as a powerful tool that can aid in the progress of finding new pharmacological treatments.

## Introduction

ALS is a complex and currently uncurable neurodegenerative disease that affects both upper and lower motor neurons (MNs). Although considered rare, it is the most common motor neuron disease, with an incidence of 1–2 cases per 100.000 people per year (Logroscino and Piccininni, 2019). This pathology presents a late-onset, with an average of 50–60 years of age, that progresses from muscle weakness towards the patient’s eventual decease due to respiratory muscle failure (Bruijn et al., 2004; Pasinelli and Brown, 2006). It is estimated that 90% of ALS cases have an unspecific etiology, and thus are categorized as apparently sporadic ALS (sALS). Familial ALS (fALS), which represents the remaining 10% of cases, is defined by specific genetic alterations and traced through the patient’s family history. Both categories, although widely used, can be biased and unreliable in their distinction (Brown and Al-Chalabi, 2017; Turner et al., 2017). Factors such as unknown family history, non-paternity and incomplete genetic penetrance, contribute to this misclassification (Robberecht and Philips, 2013). Lastly, the disease’s complexity, due to symptoms’ heterogeneity -rate of progression, onset site, and the presence of various degrees of cognitive dysfunction- confers further difficulties in providing effective treatment.

ALS patients’ life expectancy is about 3–5 years from symptoms onset, with current drug treatments providing limited improvement for select patients (Brown and Al-Chalabi, 2017). In this sense, therapeutic and pharmacological advances have been slow, mainly attributed to the limitations in the disease’s modeling. Neuropathological tissue samples are difficult to obtain, and restricted to rare invasive biopsies and *post-mortem* tissues (Stan et al., 2006). Furthermore, they model the final stage of the disease and therefore are unreliable for studying its progression. while animal models are useful to elucidate some of the disease’s underlying mechanisms, and most interestingly, to observe their behavioral cues, their translational value for drug development is yet to be proven (Van Norman, 2019). The intrinsic differences between species, including immunological response and anatomical structures, such as the absence of neurological disease-relevant tissues (Eaton and Wishart, 2017) contribute to their limitations. Furthermore, animal models can only replicate hereditable traits, excluding sporadic forms that, as mentioned, represents most diagnosed cases. In this context, the introduction of iPSC technology has allowed an unprecedented opportunity to model nervous system diseases, from both genetic and idiopathic origin. We are certain that advances in 3D cell culture systems coupled with the integration of bioinformatics tools will considerably change our current understanding and therapeutic landscape of ALS.

### Amyotrophic lateral sclerosis molecular and genetic characterization

In broad terms, the pathological hallmark of ALS involves the degeneration of MNs of the motor cortex, spinal anterior horn, and the lateral columns of the spinal cord (Saberi et al., 2015). Patients with ALS exhibit both upper and lower MN disease symptoms such as muscle weakness, hyper reflexivity, spasticity, and/or rigidity (Redler and Dokholyan, 2012). Patients have also reported symptoms involving exercise intolerance and cognitive impairment (Mezzani et al., 2012; Chiò et al., 2017; Cividini et al., 2021). The inexorable progression of muscle weakness leading towards disability, combined with cognitive changes (Phukan et al., 2007), is devastating for both patient and loved ones. Due to the severe impact of the disease, there is an urgent need to understand the genetic and molecular underpinnings of ALS.

From decades of research dedicated to uncovering the genetic basis of ALS, researchers have discovered over 50 genes to be potentially causative or disease-modifying (Mejzini et al., 2019). The major genes described for ALS include *C9orf72, SOD1, TARDBP, and FUS*. The *C9orf72* gene contains a hexanucleotide repeat expansion of the six-letter string of nucleotides GGGGCC. The normal length of the repeat consists of about 2–24 nucleotides, whereas mutation carriers have more than 30 and even up to hundreds or thousands of repeats (Smeyers et al., 2021). *C9orf72* accounts for about 33% of fALS and less than 5% of sALS cases in European populations (Zou et al., 2017). *SOD1* was the first gene discovered for fALS and codes for superoxide dismutase 1. Although the role of SOD1 is heavily debated, there is evidence for a toxic gain-of-function (Healy et al., 2020). Mutations in *SOD1* account for about 30% of fALS, and 2–7% of sALS cases (Bernard et al., 2020). TDP-43 is encoded by the *TARDBP* gene and is a transcriptional repressor. *TARDBP* mutations only account for about 3% of fALS (Rutherford et al., 2008). Finally, *FUS* encodes an RNA binding protein with the mutations accounting for about 1% of fALS and less than 1% of sALS. *FUS* mutations are associated with juvenile ALS presenting basophilic inclusions (Picher-Martel et al., 2020).

The genes described above share and differ in the disrupted cellular processes. *C9orf72, SOD1, TARDBP,* and *FUS* mutations are associated with impaired proteostasis, but other processes, such as cytoskeleton and axon-transport defects, are associated with *SOD1,* while disturbed RNA metabolism is seen in *TARDBP* and *FUS*. There are several other genes with known disease-significance, such as TP73 (Russell et al., 2021), which is implicated in apoptosis (Wood, 2021).

The identification of new genes and mutations is increasing due to technological advances and increased awareness. With the great number of genes implicated in the disease, and even greater number of mutations associated with each gene, it becomes a challenging task to unveil the critical pathological mechanism of ALS. A characteristic molecular feature is the presence of cytoplasmic inclusions. For example, TDP-43 is a protein present in these inclusions, which are shared between both sALS and fALS (Gruzman et al., 2007; Prasad et al., 2019). In this sense, deciphering the mechanisms of protein misfolding and the role of misfolded protein is key to understanding pivotal aspects of ALS pathomechanisms. Interestingly, recent research has attributed soluble misfolded proteins as the toxic player in neurodegeneration rather than the large aggregates (Kirkitadze et al., 2002; Urushitani et al., 2006; Proctor et al., 2016; Choi and Dokholyan, 2021), implying a protective mechanistic role of large aggregates (Zhu et al., 2018). The toxic mechanism of soluble misfolded proteins is unknown, but major cellular processes include dysfunctional RNA metabolism, impaired proteostasis, mitochondrial dysfunction, and excitotoxicity (Ruegsegger and Saxena, 2016; Butti and Patten, 2019; Calió et al., 2020; Gunes et al., 2020). With the complexity of the aberrant cellular processes and unknown disease triggers, it poses a challenge for developing an effective, common therapeutic.

Historically, neurological disease research focused on neurons as a single and isolated functioning unit. However, dismissing the complex interplay of the stroma and other disease-implicated cell types results in a lack of physiological relevance, more so when modeling a systemic disease. ALS possesses great genotypic and phenotypic heterogeneity, where both cell-autonomous and non-cell-autonomous factors drive the diseases’ progression towards common clinical manifestations. MNs and glial cells are in continuous interaction in order to maintain proper tissue homeostasis. Among other functions, glial cells provide trophic factors, myelinate axons, and clear cell debris, that is, they are critical for health maintenance and survival. Transcriptomics data revealed that multiple non-neuronal cell types are vulnerable to dysfunction and are crucial to maintaining neuronal health (Enright et al., 2020; Gleichman and Carmichael, 2020). Glial cells have been implicated in the initiation of ALS, and its progression (Brites and Vaz, 2014). In addition, astrocytes from SOD1 mice are sufficient to kill motor neurons in WT mice supporting non-cell autonomous effects (Nagai et al., 2007) iPSC derived astrocytes from ALS patients showed impaired autophagy through non-cell-autonomous mechanisms (Madill et al., 2017). iPSCs and co-cultures are potentially beneficial to incorporate critical controllers of neuronal health into ALS studies. This involvement of non-neuronal cell types is consistent with the non-cell-autonomous theory that challenges the cell-autonomous theory (Chen et al., 2018; Argueti-Ostrovsky et al., 2021).

The complexity of ALS, evidenced by the number of cellular aberrant processes and its heterogeneity indicates that the disease is most likely caused by multiple factors rather than a single inciting event (Chiò et al., 2018; Mejzini et al., 2019). Improving models of disease by using iPSCs and 3D-cultures can aid in highlighting the most critical cellular processes disturbed in ALS disease pathogenesis and thus, allow for the discovery of new therapeutic compounds.

### Overview of induced pluripotent stem cells technology and culture characterization

Briefly, in 2006, Takahashi and Yamanaka reported the development of iPSCs, via retroviral transduction of four transcription factors (OCT3/4, SOX2, KLF4, and c-MYC) delivered into mouse fibroblasts. These cells were characterized as embryonic stem cells (ESCs)-like concerning their morphology, growth pattern, ability to differentiate, express pluripotent cell markers, and form teratomas after being injected into immunodeficient mice (Takahashi and Yamanaka, 2006). Shortly after, iPSCs generated from human somatic cells were introduced (Takahashi et al., 2007; Wernig et al., 2007; Yu et al., 2007; Kim et al., 2008; Park et al., 2008). Most importantly, these cells bypass ethical concerns associated with the destruction of human embryos, and are easily obtained by avoiding invasive procedures (Zarzeczny et al., 2009; Niemiec and Howard, 2020). Furthermore, as *bona fide* stem cells, their self-renewal capabilities offer a robust supply of unlimited patient-derived cells, which is especially relevant, as the rare-to-obtain neural tissue samples lack the cell quantity needed for drug screening assays. However, there are some disadvantages with their reprogramming efficiency, being as low as 0.1–1% (Mali et al., 2010), and the presence of incomplete reprogrammed cell phenotypes, epigenetic memory, and genomic instability (Mikkelsen et al., 2008; Kim K. et al., 2010; Gore et al., 2011; Liu et al., 2020).

**FIGURE 1:**
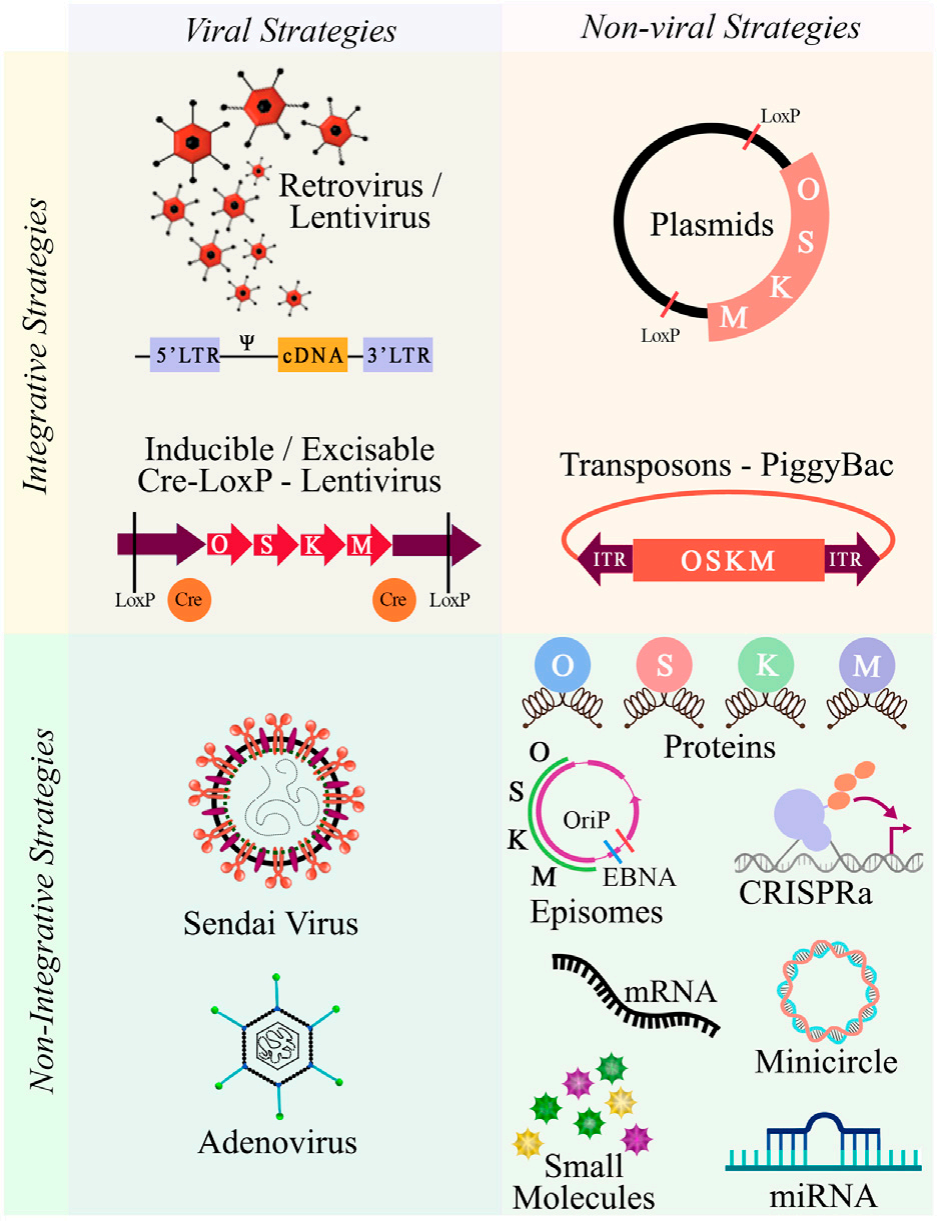
Integrative and non-integrative reprogramming strategies. Schematic overview of the available methodologies to dedifferentiate cells towards pluripotency. Abbreviations: O, OCT3/4; S, SOX2; K, KLF4; M, c-MYC.

To obtain iPSCs, it is necessary to select an adequate reprogramming cell population, as not all cell types have shown the same reprogramming efficiency, mostly attributed to their characteristic gene expression profile and epigenetic status (Liebau et al., 2013). An attractive cell source is peripheral blood mononuclear cells (PBMCs), on account of both, the non-invasiveness of the extraction procedure and their reprogramming efficiency (Trokovic et al., 2014; Ye and Wang, 2018). The next step is choosing the right combination of reprogramming factors, given that they can be endogenously expressed in some cell types (Kim J. B. et al., 2009). Optionally, reprogramming enhancers can be added (reviewed by Kwon et al., 2017). The nature of the research and technical skills of the laboratory will condition the selection of the reprogramming strategy. Although there are several available methods, not all are deemed suitable for translational medicine. Two broad strategies can be undertaken, an integrative strategy, which as the name suggests, involves the integration of exogenous genetic material, and a non-integrative strategy, also known as footprint free, which uses excisable vectors or molecules that do not integrate into the host genome. The advantage of the first strategy is the persistent expression of the reprogramming factors, however, there are high risks of genomic instability, random integration leading to insertional mutagenesis, reactive transgenes and chromosomal aberrations (Nakagawa et al., 2008; Tong et al., 2011; Takahashi and Yamanaka, 2013). Both viral and non-viral delivery methods are found within this strategy. The viral methods include the classical use of retrovirus (Takahashi and Yamanaka, 2006) and lentivirus (Yu et al., 2007). The non-viral methods include transfecting plasmids (Okita et al., 2008) and transposons (Woltjen et al., 2009). Within the non-integrative strategies, clinical-grade and translational suitable iPSCs can be obtained, mainly through the direct delivery of microRNAs (Anokye-Danso et al., 2011; Kogut et al., 2018), mRNAs (Warren et al., 2010; Yakubov et al., 2010), episomes (Yu et al., 2009), cell-penetrating peptides (Kim D. et al., 2009), small molecules (Hou et al., 2013), and viral vector transduction via sendai virus (SeV) (Fusaki et al., 2009) or adenovirus (Zhou and Freed, 2009). More recently, a CRISPR-Cas9 variant, CRISPR activation (CRISPRa) has been introduced as a reprogramming tool (Weltner et al., 2018) (Figure 1). Some of these non-integrative systems have combined both quality and safety standards by introducing xeno-free culture methods that meet good manufacturing practice standards (GMP) (Chen et al., 2011; Wang et al., 2015). Do to the robustness of the reprogramming process, it is suggested that the chosen method does not significantly affect the differentiation potential of the produced iPSCs (Churko et al., 2017).

Once iPSCs cultures are obtained, their characterization must be performed through a combination of stringent analysis that ensures their translational value, quality, and reproducibility. A quick indicator is their morphology, which should be consistently observed as tightly packed colonies, with smooth and defined borders, composed of small cells with a large nucleus/cytoplasm ratio (Meissner et al., 2007; Takahashi et al., 2007). Regular monitoring of genotoxicity is unavoidable, since genomic alterations are attributed to the effects of the reprogramming method, prolong cell culture, and pluripotency-induction itself (Hong et al., 2013; Liu et al., 2020). Moreover, controls to evaluate the presence of residual exogenous reprogramming factors, footprint, or inefficient plasmid clearance are needed, as it hinders the cells capabilities to differentiate, and predispose them to genomic instability (Yu et al., 2007; Ramos-Mejia et al., 2010; Sommer et al., 2012).

Regarding molecular characterization, robust test-sets often include detection of core-factors and proteins related to pluripotent stem cells (PSCs) and ESCs, such as NANOG, OCT4, SOX2, tumor-related antigen (TRA)-1-60/81, stage-specific embryonic antigen (SSEA)-3/4, and the traditional staining of alkaline phosphatase (ALP) (Boulting et al., 2011).

**FIGURE 2:**
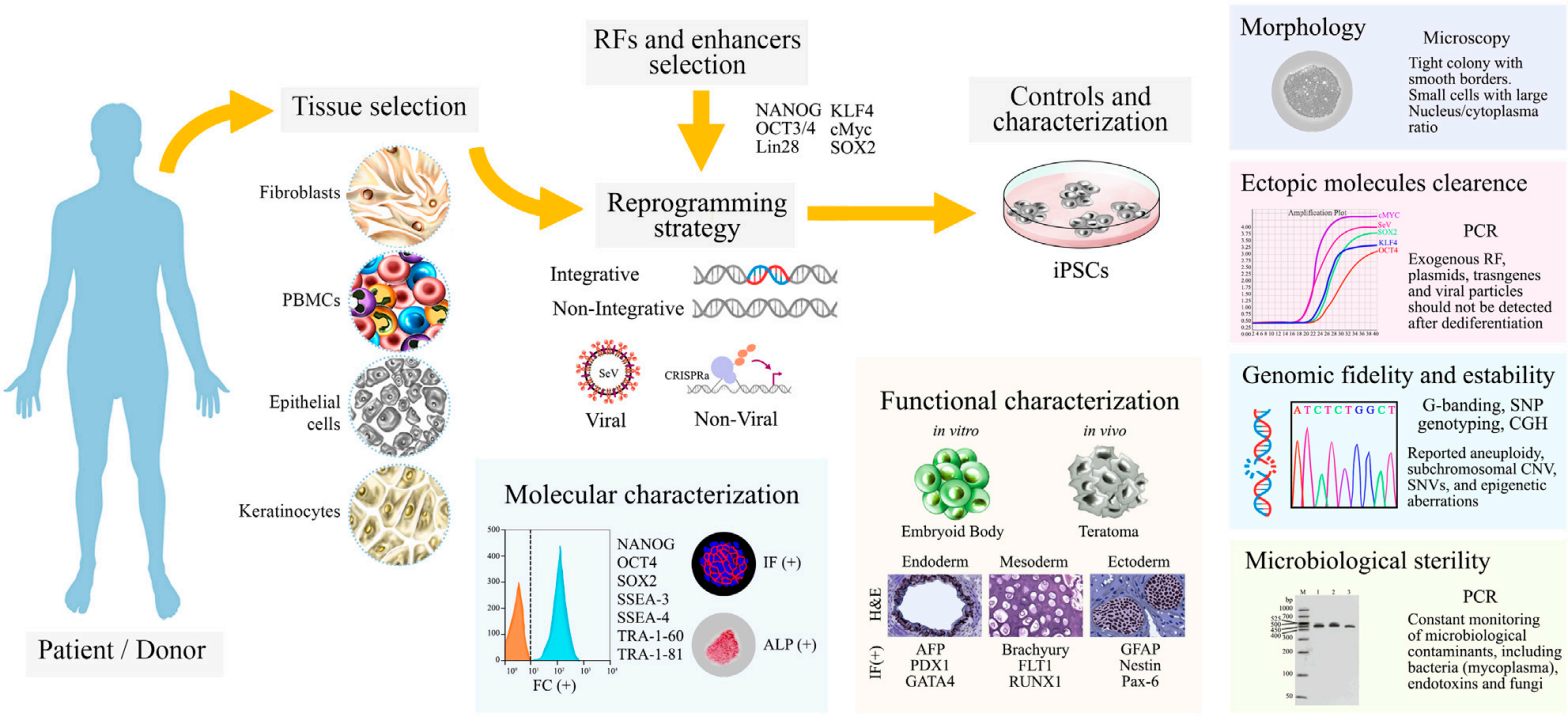
A schematic overview from patient’s tissue sample to characterized iPSCs cultures. Patient/donor samples are chosen, the four most frequent tissues are fibroblasts, blood cells, urine track epithelial cells and keratinocytes. Reprogramming factors (RFs), enhancers, and the reprogramming strategy are carefully selected. Once iPSCs cultures are obtained, controls and characterization assays are performed as shown. Abbreviations: SeV, Sendai virus; CRISPRa, CRISPR-Cas9-based gene activation; IF, immunofluorescence; ALP, alkaline phosphatase assay; H&E, hematoxylin and eosin; SNP, single-nucleotide polymorphism; CGH, comparative genomic hybridization; CNV, copy number variation.

Furthermore, functional assays must demonstrate their pluripotency by differentiating toward cells belonging to the three-germ layers. Both *in vitro* and *in vivo* approaches can be undertaken. The *in vitro* approach includes molecule-based differentiation or spontaneous embryoid body (EB) generation (Sheridan et al., 2012), while the *in vivo* approach involves teratoma formation (Müller et al., 2010). Regardless of the method, cell lineage identity is assessed by markers such as GFAP, NESTIN, PAX6 (Ectoderm); AFP, PDX1, GATA4 (Endoderm); and Brachyury (TBXT), FLT1, RUNX1, FOXA2 (Mesoderm) (Figure 2).

Obtaining iPSCs is a laborious and costly process that also requires significant expert training. As experts in the field will agree, high-quality iPSCs will determine the effectiveness and efficiency of the subsequential differentiation process. In this sense, we believe in the importance of incentivizing and joining efforts to establish cell repositories or comprehensive cell libraries. Publicly available and fully characterized iPSCs derived from diverse ALS genetic backgrounds and idiopathic origins can immensely aid the scientific community (Li et al., 2015).

### The un-standardized journey, from iPSCs to motor neurons

iPSC technology, coupled with a deeper understanding of the ectodermal cell differentiation process has allowed for the production of diverse ALS neural cell types. This includes MNs (Dimos et al., 2008), oligodendrocytes (OLGs) (Ferraiuolo et al., 2016), astrocytes (ACs) (Birger et al., 2019), sensory neurons (SNs) and microglia cells (Pereira et al., 2021). Since multiple pathological mechanisms, both genetic and molecular, converge in the degeneration and death of MNs, most of the published works focus on obtaining and characterizing these MN mono-culture systems.

In broad terms, molecule-based differentiation process recapitulates neurodevelopment. Cells transition towards the ectoderm layer, where both neural stem cells (NSCs) and neural progenitor cells (NPSCs) can be specified by supplementing cultures with appropriate molecules that regulate cell-identity. Current protocols show large variations (reviewed by Faravelli et al., 2014; Sances et al., 2016), however, they all rely on the same stages: induction of neuralization, caudal patterning, ventral patterning, and a final maturation stage.

**FIGURE 3:**
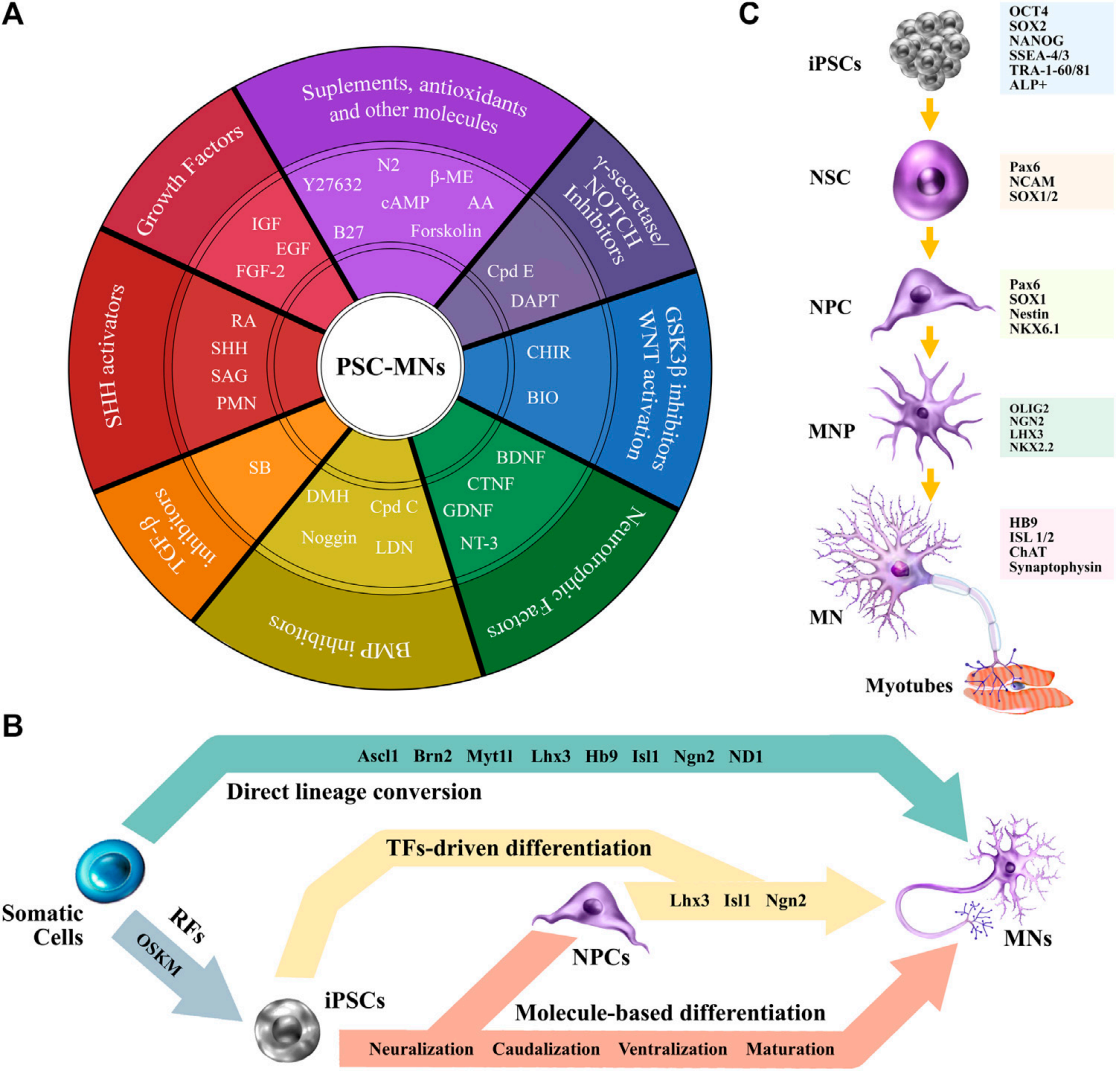
From PSCs to MNs. **(A)** A schematic view of commonly used molecules to differentiate PSCs towards MNs. **(B)** Differentiation strategies. **(C)** iPSCs-MN cell markers for characterization. Abbreviations: RFs, reprograming factors; TFs, transcription factors; ß-ME, ß-Mercaptoethanol; AA, ascorbic acid; Cpd E, compound E; cAMP, cyclic adenosine monophosphate; DAPT, N-[(3,5-difluorophenyl)acetyl]-l-alanyl-2- phenyl]glycine-1,1-dimethylethyl ester; GSK3, glycogen synthase kinase 3; WNT, wingless/integrated; CHIR, CHIR99021; BIO, (2′Z, 3′E)-6-bromoin-dirubin-3′-oxime; BDNF, brain-derived neurotrophic factor; CNTF, ciliary neurotrophic factor; GDNF, glial-derived neurotrophic factor; NT-3, neurotrophin-3; BMP, bone morphogen protein; Cpd C, compound C; LDN, LDN193189; RA, retinoic acid; DMH, dorsomorphin; SHH, sonic hedgehog; TGF-ß, transforming growth factor-ß; SB, SB431542; SAG, smoothened agonist; IGF, insulin-like growth factor; FGF-2, fibroblast growth factor-2; EGF, epidermal growth factor; PMN, purmorphamine.

Neural induction results in the generation of NPCs, parting either from EBs or monolayer cultures, and involves the regulation of several signaling pathways, including the fibroblast growth factor (FGF), bone morphogen protein (BMP), transforming growth factor-β (TGF-β), and notch pathway (Joannides et al., 2007; Du et al., 2015; Maury et al., 2015; Bianchi et al., 2018). The Neuralization process is commonly performed by the synergistic action of dual-SMAD inhibitors (SMADi), comprising SB431542 (SB) -a nodal inhibitor, member of the TGF-β signaling pathway- and LDN193189 (LDN) or noggin -a BMP inhibitor- (Chambers et al., 2009; Fukusumi et al., 2016). Moreover, the efficiency of this process is further increased when combining SMADi with γ-secretase inhibitors and WNT activators (Bianchi et al., 2018). The next stage involves caudalization, which is mainly driven by the effects of retinoic acid (RA) (Li et al., 2005; Qu et al., 2014). The activation of WNT/β-catenin signaling *via* GSK3β inhibition, either by (2’Z, 3’E)-6-bromoin-dirubin-3’-oxime (BIO) or CHIR99021 (CHIR) optimizes the procedure (Maury et al., 2015; Shimojo et al., 2015). Ventral patterning is orchestrated by a key Smoothened (Smo) agonist, sonic hedgehog (SHH) (Li et al., 2005), or its synthetic alternatives, purmorphamine (PMN) and SAG (Wichterle et al., 2002). Finally, maturation can be guided by neurotrophic factors, including insulin-like growth factor-1 (IGF-1), glial-derived neurotrophic factor (GDNF), and brain-derived neurotrophic factor (BDNF) (Li et al., 2005; Hu and Zhang, 2009; Burkhardt et al., 2013). Lastly, the γ-secretase inhibitor, compound E (Cpd E), displays capabilities to enhance and shorten maturation time (Du et al., 2015) (Figure 3A).

Of great importance, Maury et al. (2015) demonstrated how fine tuning of the combination, concentration, and exposure time of the differentiating factors impact cell specification. Proving that these subtle modifications cue MN progenitors (MNPs) to differentially specify four neuronal subtypes, namely, interneurons, SNs, cranial MNs, and spinal MNs.

Highly relevant to both translational and clinical research is the development of GMP protocols performed under fully humanized conditions. To our knowledge, there is only one report of a fully xeno-free protocol from iPSCs to MN production (Hu et al., 2016).

An alternative strategy to obtain iPSC-MNs involves the combination of molecule-based differentiation with transcriptional coding. After neuralization of iPSCs, NPCs can be transduced with LIM/homeobox protein 3 (Lhx3), Islet-1 (Isl1), and neurogenin 2 (Ngn2) -LIN, either via adenovirus (Hester et al., 2011) or lentivirus (Sepehrimanesh and Ding, 2020). Other protocols bypass neuralization and force the expression of the transcriptional factors directly into iPSCs (Figure 3B). As an example, lower-MNs are obtained by generating LIN-inducible transgenic lines (Fernandopulle et al., 2018). Footprint-free approaches, that avoid genomic integrity concerns, use SeV encoding LIN (Goto et al., 2017), piggyBac transposon vectors (Imamura et al., 2017), and synthetic-mRNA encoding Ngn1, Nng2, Ngn3, NeuroD1 (ND1) and ND2 (Goparaju et al., 2017). Cranial MNs have also been obtained via the ectopic expression of Ngn2, Isl1, and Phox2a (Garone et al., 2019). Recent work shows that overexpression of just Ngn2 coupled with molecular patterning of RA and SAG produces a pure population of lower induced MNs (LiMoNe). LiMoNe cells are obtained with a high-yield, and display both electrophysiological activity and form synaptic contacts with muscle cells (Limone et al., 2022).

Transcriptional factor-driven differentiation shows some advantages over molecule-based differentiation, including an overall reduction in time, cost and technical skills, as well as an increase in culture purity (reviewed by Canals et al., 2021). Moreover, molecule-based differentiation generates cultures that contain mixed-degrees of differentiated cells, which while advantageous as a developmental model, its a concern for drug screening. Therefore, MN-enrichment may be required as an additional step. For this, protocols include gradient centrifugation (Corti et al., 2012), magnetic cell sorting of L1CAM (CD171) (Maciel et al., 2018), and more recently, fluorescent activated cell sorting (Klim et al., 2019). This last strategy allows for efficient enrichment of post-mitotic *Hb9*::GFP + MNs based on their NCAM+/EpCAM-immunoprofile.

A direct lineage conversion strategy has been refined, which involves bypassing pluripotent reprogramming, and directly converting somatic cells into other lineages. The process centers on forced expression of transcriptional factors by retroviral transduction. The combination of factors includes Ascl1, Brn2 (also known as Pou3f2), Myt1l, Lhx3, Hb9, Isl1, Ngn2, and ND1. The resulting cells, coined *induced* MNs (iMNs), showed electrophysiological activity, neuromuscular junction (NMJ) formation, and molecular and functional properties of naïve MNs (Son et al., 2011). Induced Neurons (iNs) can also be produced in this manner by lentiviral infection of Brn2, Ascl1, Myt1l, and ND1 -BAMD. However, these cells did not exhibit a fully mature phenotype (Pang et al., 2011). Notably, there are some drawbacks to this methodology, including their limited yield, as no stem cell nor progenitor cell stages are present, therefore their number is restricted to the parting cell count. Moreover, there is evidence that transdifferentiation efficiency decreases in virtue of the donor’s age (Montana et al., 2013), which becomes an obstacle for modeling late-onset diseases. Furthermore, the use of integrative-viral strategies, as previously mentioned, poses risk of insertional mutagenesis.

As a cautionary tale, both the dedifferentiation and differentiation processes subject cells to metabolic and epigenetic changes that can lead to genomic variations. In addition to this, protocol diversity, un-standardized cell culture technique, and intrinsic variables associated with the cells *per-se*, contribute to cell culture heterogeneity. Furthermore, PSC lines have shown variating innate degrees of differentiation potential towards a determined cell lineage, attributable to their individual genetic and epigenetic background (Osafune et al., 2008). Remarkably, a strategy to coax iPSCs lines with unsimilar differentiation propensity towards a neural lineage is achieved by SMADi, which is already an established stage of the MN differentiation protocol (Kim D.-S. et al., 2010).

Regardless of the differentiation strategy, cell characterization is central for appropriate downstream research and data analysis. Early neuroectodermal markers such as Pax6, NCAM, and SOX1/2 can be used to identify NSCs (Joannides et al., 2007). For NPCs, there is overlapping of markers, like Pax6 (Chambers et al., 2009), and SOX1 (Bianchi et al., 2018), that can be used along with Nestin and Nkx6.1 (Maury et al., 2015). MNP markers include further overlapping of Pax6, and the increasing expression of Olig2, Ngn2, Lhx3, NKx2.2 (Hu and Zhang, 2009; Maury et al., 2015; Shimojo et al., 2015; Bianchi et al., 2018). Mature MN markers comprise HB9, Isl1/2, C*h*AT (Hu and Zhang, 2009; Maury et al., 2015), and Synaptophysin, an indicator of possible synaptic connectivity (Bianchi et al., 2018) (Figure 3C). Cytoskeletal pan-neural markers, MAP2 (dendrite), *ß*-III tubulin [(Tuj1, TUBB2); axon, dendrite and Soma], and neurofilament (axon) (Qu et al., 2014; Bianchi et al., 2018; Sepehrimanesh and Ding, 2020) are also frequently used. The absence of proliferative markers, as Ki67, can further characterize cells in their post-mitotic stages (Maury et al., 2015).

Functional characterization validates the system in study and offers physiological relevance to the generated data. Thus, MNs should be synaptically mature, and exhibit an appropriate electrophysiological activity. Several strategies can be used to assess these characteristics, including patch-clamp, calcium imaging (Prè et al., 2014), optopatch (Kiskinis et al., 2018) and multielectrode array (MEA) (Taga et al., 2021). A central MN ability, indicative of a mature phenotype, is NMJ formation. These are specialized cholinergic synapsis that transmits chemical signals to muscle fibers from the electrical impulse generated by MNs. They can be identified by α-BTX staining of acetylcholine receptors (AChRs) clustering on myotubes juxtaposed to axon terminals (Lin et al., 2019).

Thus far, we have overviewed the process to obtain iPSC-MNs from donor cells samples in the interest of introducing their use in drug screening, and discuss how 3D-modeling and bioinformatics can aid ALS research.

### Induced pluripotent stem cell derived from ALS patients as drug screening models

A significant interest in iPSC-ALS modeling is associated with targeting both fALS and sALS (Fujimori et al., 2018; Sun et al., 2018). However, the diversity of the reprogramming methods (Guo et al., 2017a), characterization criteria and standards, and protocols to re-differentiate cells towards neuronal lineages (Hawrot et al., 2020) hinders appropriate comparison of results between the generated data. A crucial tool for increasing confidence in iPSC-ALS modeling is the use of isogenic lineages. These lineages are obtained either from correcting the mutation of the parental-line in study, or by inserting a mutation that correlates with the disease’s phenotype. These cultures should only differ in the genome-edited loci, and can be performed by CRISPR (Deneault et al., 2021), transcription activator-like effector nucleases (TALENs) (Chen et al., 2014), and zinc-finger nucleases (ZFNs) (Kiskinis et al., 2014). Unfortunately, it is not possible to produce isogenic controls when the genetic lesion is not identified, as in some cases of sALS. The lack of isogenic lineages difficult the discerning of the disease’s phenotype from the cells own phenotype. Relevant to ALS drug screening is a novel scalable platform based on CRISPR interference (CRISPRi). This technology centers on robust gene-knockdown that allows for functional characterization of genes that control disease-relevant phenotypes including neuron survival and morphology. Importantly, it enables interrogation of ‘druggable genome’. This technology coupled with isogenic controls could elucidate new disease mechanisms that, in turn, lead to the discovery of novel drug-targets (Tian et al., 2019).

**FIGURE 4:**
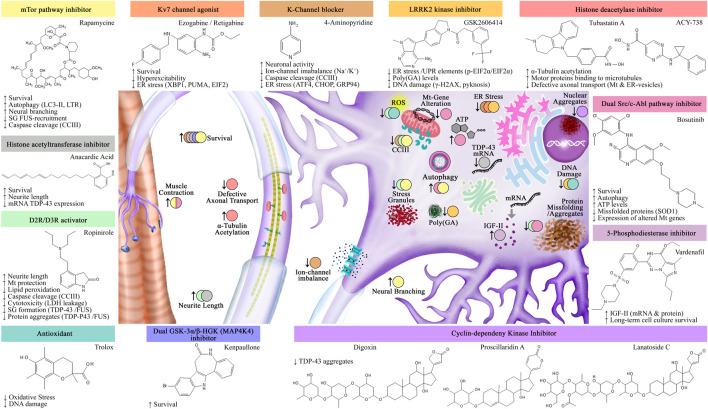
Assessed drugs using iPSC-ALS modeling. Identified drugs and their effects in both iPSCs-MNs and muscle fibers. Abbreviations: SG: stress granules; Mt: mitochondria; ER: endoplasmic reticulum; UPR: unfolded protein response.

Recent reviews have centered on the use of iPSCs-based drug platforms for neurodegenerative diseases (reviewed by Bonaventura et al., 2021; Ferraiuolo and Maragakis, 2021; Pasteuning-Vuhman et al., 2021) Specifically for ALS, researched drugs include rapamycin (Imamura et al., 2017; Marrone et al., 2018; Osaki et al., 2018), bosutinib (Imamura et al., 2017; Osaki et al., 2018), anacardic acid (Egawa et al., 2012), ropinirole (Fujimori et al., 2018), vardenafil (Osborn et al., 2018), ezogabine (Wainger et al., 2014), GKS2606414 (Szebényi et al., 2021), trolox (Lopez-Gonzalez et al., 2016), 4-aminopyridine (Naujock et al., 2016), kenpaullone (Yang et al., 2013), the cyclin-dependent kinase inhibitors: digoxin, lanatoside C, and proscillaridin (Burkhardt et al., 2013), and the histone deacetylation inhibitors: HDAC6 antisense oligonucleotide-ASO, tubastatin A and ACY-738 (Guo et al., 2017b) (Figure 4). In the limelight, three of these drugs, ropinirole (Morimoto et al., 2019), retigabine (Wainger et al., 2021), and bosutinib (Imamura et al., 2019) are currently in clinical trial.

Two concerns are put forward, the first involves cell-aging or the conservation of the epigenetic drift in iPSCs, as ALS is a late-occurring disease (Pandya and Patani, 2020). Several authors have evidenced iPSCs rejuvenation by their whole-transcriptome RNA sequencing (RNA-seq) (Mertens et al., 2015), nuclear organization, reduced DNA damage, mitochondrial metabolism (Miller et al., 2013), telomere length (Agarwal et al., 2010), and the loss of senescence markers (Lapasset et al., 2011). To overcome this age-setback, artificially induced-aging strategies have been developed. For instance, telomerase inhibition (Vera et al., 2016), and the forced ectopic expression of progerin, a truncated form of the nuclear envelope protein laminin A (Miller et al., 2013). This last molecule was able to phenocopy tissue-specific age-associated features that were later corroborated *in vivo* (Miller et al., 2013). However, much uncertainty remains as to the degree with which age-related physiology is mimicked.

The second concern involves the possible loss of non-genetic disease-related traits, given that, during the reprogramming process, cells suffer epigenetic reshaping allowing cell-identity pattern erasing. Analogously, the possibility of acquiring unknown and disease-unrelated features can render the model less relevant (Liang and Zhang, 2013). Assessment of DNA-methylation and histone marks from the reprogrammed iPSCs to the patients’ wild-type cells, as well as the use of isogenic controls increases the model’s credibility.

Direct conversion could, to some extent, breach both aging and epigenetic concerns. In this sense, iNs from donors of diverse ages presented age-correlated features including transcriptomic signature, compromised nucleocytoplasmic compartmentalization (Mertens et al., 2015), microRNA profile, and DNA-methylation status (Huh et al., 2016). Aging hallmark preservation has also been observed in iMNs. Nuclear organization (heterochromatin loss and DNA damage), and increased senescence-activated *ß*-galactosidase activity (Tang et al., 2017) have been reported. However, direct conversion advantages related to cost, time, and epigenetic status should be carefully considered in relation to their limitations. These limitations include culture scale-up and their ability to form organoids.

### Three-dimensional cultures

Variations in cellular behavior, drug response and pathomechanisms can be detected according to the cell culture system used. Three-dimensional (3D) systems are regarded with higher predictive value as they encompass diverse cell type interactions, ECM interplay, and complex microenvironmental cues not found in two-dimension (2D) cultures (Pampaloni et al., 2007; Maltman and Przyborski, 2010).

A wide variety of 3D-culture systems have been implemented in neurosciences. Complex tissue constructs, such as brain region-specific organoids, including cerebral (Lancaster and Knoblich, 2014), midbrain (Nickels et al., 2020), forebrain (Gomes et al., 2020), hypothalamus (Qian et al., 2018), and cerebellum (Muguruma et al., 2015), as well as spinal cord organoids (Hor et al., 2018), spheroids (Paşca et al., 2015; Bowser and Moore, 2019), assembloids (Birey et al., 2017), and brain-on-a-chip technology (Adriani et al., 2017) are on the rise.

A critical component in the generation of these tissues is the presence or absence of a scaffold. The scaffold-free approach involves spontaneously formed and self-organizing multilayer cell aggregates that can eventually produce their own non-cellular elements (Fennema et al., 2013; Raja et al., 2016; Türker et al., 2018). The scaffold-based approach uses a substrate that guides cell behavior and organization through mechanical and chemical cues, allowing the generation of bigger and more complex structures. These substrates are hydrogels derived from a biological source (e.g., Matrigel, Collagen, Gelatin, Alginate, etc.), have a synthetic origin (e.g., Polyglycol Acid, Polyethylene Glycol), or present a combination of both. As each material has its own limitations and advantages, the nature of the study or end-product will help determine the appropriate approach (Reviewed: Murphy et al., 2017; Rey et al., 2020; Roth et al., 2021). Noteworthy, enhanced long-term culture and mature phenotypes of neural cell types have been associated with the use of natural scaffolds, such as Hyaluronan Acid (Zhang et al., 2016; Wu et al., 2017) and decellularized brain-derived ECM (Sood et al., 2019). However, these can compromise xeno-free systems and contribute to variability, as they present batch-to-batch variations in their composition.

Although MNs are predominantly studied, ALS is a systemic non-autonomous and multicellular disease, that cannot be fully represented by a single monolayer-cell lineage. In this sense, 3D-cultures allow the formation of neural networks, with brain-like functions, and multidirectional communication between, for example, neurons and glial cells (Lancaster et al., 2013; Sakaguchi et al., 2019; Yakoub, 2019; Samarasinghe et al., 2021). However, to date, only five 3D-systems have been published for the study of ALS (Table 1).

**TABLE 1:** 3D-culture models implemented in the study of ALS.

Objective	3D-system	Cell types identified/added to the system	ALS model	Central findings	References
NMJ modeling and drug testing (Rapamycin/Bosutinib)	Microfluidic device (ALS-on-a-chip)	iPSC-ALS-MNs	sALS (TDP-43)	- iPSC-ALS motor units generated fewer contractions and NMJ formation	Osaki et al. (2018)
		iPSC-ALS-ACs		- iPSC-ALS-MN degradation	
		iPSC-SMCs	Non-ALS	- Apoptosis of iPSC-SMCs	
		iPSC-ECs		- Drug combination improved iPSC-ALS-MN survival and muscle contraction	
NMJ modeling	Sensorimotor organoid	iPSC-ALS-MNs	- fALS (C9orf72 and FUS)	- Reduced contraction	Pereira et al. (2021)
		iPSC-ALS-SNs		- Abnormal NMJs	
		iPSC-ALS-ACs			
		iPSC-ALS-Microglia			
		iPSC-ALS-SMCs	- sALS		
		iPSC-ALS-ECs			
Model development	Polystyrene scaffold 3D-culture plates (Alvetex, Reprocell)	iPSC-ALS-Cortex neurons	fALS (C9orf72)	- Spontaneous cell cycle protein expression	Porterfield et al. (2020)
		iPSC-ALS-ACs		- Senescence-associated gene expression and protein secretion	
Drug testing (Rapamycin)	Microfluidic device (Drug gradient control)	mESC-ALS-MNs	Transgenic mouse (TDP-43-A315T)	- MN’s survival increased in a dosage-dependent manner	Chennampally et al. (2021)
Model development and drug testing (GSK 2606414)	Cerebral cortical organoid slice	iPSC-ALS-ULNs	ALS (C9orf72)	- Astroglia and neurons present transcriptional and proteostasis disturbance-P26 and DPR poly (GA)—GKS2606414 treatment reduces cell damage	Szebényi et al. (2021)
		iPSC-ALS-DLNs			
		iPSC-ALS-IN			
		iPSC-ALS-OL/OPC			
		iPSC-ALS-iRG/oRG			
		iPSC-ALS-ACs			
		Unidentified cells and other immature/progenitors			

Abbreviations: NMJs, neuromuscular junctions; SNs, sensory neurons; ACs, astrocytes; SMCs, skeletal muscle cells; ECs, endothelial cells; mESC, mouse embryonic stem cell; ULN, upper layer cortical neuron; DLN, deep layer neuron; IN, interneurons; OL/OPC, oligodendrocyte/progenitor cells; iRG, inner radial glia; oRG, outer radial glia; CP, choroid plexus; DPR poly (GA), dipeptide repeat protein (DPR) poly (GA).

Among these systems, organ-on-a-chip, is a microfluidic device that aims to replicate the physiological microenvironment, and allows the study of NMJs between skeletal muscle cells (SMCs) and MNs (Osaki et al., 2018). NMJs are highly relevant for ALS, as its structural disassembly, and muscle denervation are both hallmarks of the disease (Cappello and Francolini, 2017). With this system, Osaki et al. were able to study iPSC-ALS-MNs phenotypical characteristics, neurite elongation speed and length, as well as the positive therapeutic effects of rapamycin and bosutinib. Thus, variables such as axonal outgrowth, synapse formation, muscle contraction, and atrophy can be assessed, highlighting the device’s appealing approach to study drug efficiency for motor unit recovery (Uzel et al., 2016; Osaki et al., 2018).

A more recent model, developed by Pereira et al. (2021), involves the use of iPSCs derived from fALS (C9orf72 and FUS) and sALS. The produced organoids were patterned towards neuromesodermal progenitors. Diverse cell types were identified in these tissue constructs, including MNs, SNs, ACs, microglia, SMCs, vasculature cells and dorsal spinal cord cell derivatives. Unlike the previous model, all cell types present in the system originate from an ALS source. Moreover, these sensorimotor organoids facilitate cell type quantification, enable live-cell imaging, and the study of NMJs, which were found impaired compared to non-pathological controls.

A third model was established using a commercially available 3D-culture plate (Alvetex, Reprocell). Porterfield et al. (2020), studied cortex neurons derived from patients presenting a C9orf72 mutation. They reported that, compared to a 2D-culture-control, phenotype-disease relevant characteristics were observed. These include, spontaneous expression of cyclin D1, dysregulation of cell cycle-associated genes, and the expression of senescence related secretory molecules*.*

With a different approach, Chennampally et al. (2021), explored the effects of rapamycin in a 3D microfluidic system that allowed for concentration-gradient control while mapping permissive zones of MN survival in the organoid. Here, the chosen disease model was derived from mouse ESCs (mESCs) harboring a TDP-43-A315T mutation. Their findings support that rapamycin dosage (0.4–1.0 µM) can increase MN survival by 40.44% through autophagy regulation of aggregates. As an advantage to the system, drug titration can be performed in a single culture, thus enhancing test throughputs and results.

The newest 3D-model, by Szebényi et al. (2021), involves the development of cortical brain organoids that are subsequently sliced and cultured on fenestrated membranes at the air-liquid interface. These organoid slices contain a consistent microarchitecture, wide cell diversity presenting forebrain signature identity, and functional and mature cortical circuits that display disease-relevant phenotypes. However, mesoderm-derived cells are not detected. The screened drug, GSK2606414, reduced unfolded protein response (UPR) activation and poly (GA) levels. Szebényi et al. (2021) suggested that dormant perinatal or pre-symptomatic cortical vulnerability is present in ALS patients.

A limitation for 3D-culture viability is their lack of vascularization, which hinders the tissue from the adequate circulation of nutrients, bioactive molecules, gaseous exchange, and waste disposal. These factors limit the organoids size, induces extensive cell death in the center or core of the tissue construct, and restricts its culture time (Lancaster et al., 2013; Grebenyuk and Ranga, 2019). However, Prolonged culture time is necessary for stem cells to differentiate and mature and mimic adult tissues. Szebényi et al. (2021) model circumvented vascularization drawback by culturing slices instead of whole organoids. These organoid slices boosted the cells viability because of increased nutrition inflow and suppressed core necrosis. In this manner, cultures were maintained for up to 240 days. However, vasculature components (e.g., ECs and pericytes) are indispensable and relevant to the appropriate differentiation and maturation of NPCs (Koutsakis and Kazanis, 2016; Cakir et al., 2019; Karakatsani et al., 2019). In this regard, two of the aforementioned 3D-models present vascular elements. Pereira et al. (2021) identified the formation of a reticulated pattern of ECs. Albeit, the group did not address its functionality nor its potential to model a blood-brain barrier (BBB), which is highly pertinent for drug screening (Cecchelli et al., 2007). While Osaki et al. (2018) model presents vascular elements, their use of a microfluidic chamber compensated—to some extent—the lack of a circulatory system. Taking a step further, they modeled a BBB-like structure, by incorporating iPSC-ECs and primming them towards a brain-specific EC-phenotype. This barrier exhibited functional characteristics by compromising the therapeutic activity of the tested drugs and allowed the authors to hypothesize its possible role in neuroprotection and muscle contraction.

The lack of standardization in 3D-cultures is a concern, and efforts to increase confidence in organoids are under way. Velasco et al. (2019), have made a major advance in developing a method to standardize individual brain organoids. They established four different protocols to generate both organoids and spheroids that were highly reproducible in cellular composition, even across different lines and batches. Single-cell sequencing confirmed that the organoids remained genetically stable along with their morphology for up to 6 months, regardless of their genetic background. Other groups have also shown reproducibility of organoids as a stable model for studies of late stages of neuronal development (Giandomenico et al., 2021), and use for neurological disorders, including ALS (Pereira et al., 2021), Alzheimer’s (Ghatak et al., 2019), and Leigh syndrome (Romero-Morales et al., 2020). The challenges ahead include consistency of the cellular and non-cellular components, the incorporation of vascular elements, immune cell, and other disease-relevant cell types (e.g., Schwann cells). As well as the development of more sophisticated scaffolds, microfluidic devices, and analysis technology adapted to 3D-systems that allow for high-throughput screening.

### The role of bioinformatics. Perspectives and future

The integration of bioinformatic and computational tools close the gap between animal and cell model disadvantages. Furthermore, allows for the understanding of the disease mechanisms by exploring large genetic datasets, identify critical biochemical pathways, and generate hypotheses. Here, we will highlight existing bioinformatic and computational tools and their applications in ALS and iPSC-ALS.

ALS genetics is complex and there is a rapidly growing dataset due to the advancement of technology and increased sampling. Existing bioinformatics tools for understanding ALS genetics are ALSoD (Abel et al., 2012) and ALSGeneScanner (Iacoangeli et al., 2019). ALSoD (Abel et al., 2012) is a freely available database, that serves as a bioinformatics repository and an analysis tool for genotype to phenotype association studies. Since the development of ALSoD, several years of repository enrichment established the evidence for the polygenic and oligogenic basis of Australian sALS (McCann et al., 2021) that would not be possible with bioinformatic analysis. In addition, ALSGeneScanner is a tool that allows non-specialists, and healthcare providers to use an automatic and annotated report for interpreting ALS genetic data. The feasibility of ALSGeneScanner allows for whole-genome and exome sequencing, variant prioritization, and helps to distinguish pathogenic from non-pathogenic variants (Iacoangeli et al., 2019). Bioinformatics has bridged complex data generated from advances in sequencing technology for ALS and continues to advance the discovery of new ALS disease-modifying genes such as *EPHA4* and *CHGB* (Pampalakis et al., 2019). The incorporation of bioinformatics provides increased approachability for non-computational experts through the accessibility of data and automatic data processing. Computational techniques, such as comparative omics, allowed researchers to find novel genetic variants such as *SPTLC1* variants and linked the variants to childhood-ALS (Mohassel et al., 2021). Most importantly, the application of bioinformatics and computational tools bolsters discoveries from genetic datasets that would otherwise take many years to connect.

For iPSCs-ALS-MNs, the application of computational techniques such as integrated multi-omic analysis resulted in the identification of novel and known aberrant cellular pathways (Li et al., 2021). Integrated multi-omic analysis distinguished pathogenic versus compensatory disease phenotype pathways, which allows for clearer understanding of disease mechanisms. In disease pathway analyses, cell type heterogeneity from iPSC cultures differentiation can confound investigations. Multi-omics integration from genomics, transcriptomics, epigenomics, and proteomics minimize variations from iPSC differentiation and highlight network-based signatures. Researchers performed comparative multi-omics analysis with selectively vulnerable MN subtypes in ALS, and using iPSCs derived from fALS and sALS patients, and have found dysregulation of lipid metabolism. Lee et al. (2021) further applied targeted metabolomics to confirm lipid metabolism dysregulation and showed pharmacological reduction of arachidonic acid reversed disease-related phenotypes in *drosophila* and mouse models of ALS (Lee et al., 2021). In this sense, bioinformatics allows for the translation of experimental studies to influence disease treatment and management.

Within mechanistic studies for ALS, errors in protein synthesis, trafficking, and degradation are regularly reported (Van Der Stappen et al., 2005). Protein studies provide insight and value in understanding the mechanism of ALS (McAlary et al., 2020). Bioinformatics aid in the study of protein-protein interactions, which are mapped and analyzed using online databases, such as STRING.db (Szklarczyk et al., 2019). STRING.db predicts not only physical protein-protein interaction but also, functional interactions. Moreover, subsequent pathway analysis is performed with both Gene Ontology (Ashburner et al., 2000) and KEGG (Kanehisa, 2000; Kanehisa et al., 2021). Gene Ontology and KEGG help to elucidate high-level functions of biological systems, identify cellular pathways and the structural locations where these occur. An example of Gene Ontology application comes from Feneberg et al. (2020), who compared human wild-type and mutant TDP-43 interactomes from iPSC-ALS, finding that disrupted protein interactions alter TDP-43 response to oxidative stress.

Beyond protein-protein interactions are cell-cell interactions that are critical for understanding disease phenotypes. The development of complex multicellular models of ALS are pushing the Frontier of *in vitro* models; however, there is a major lack in cell-cell communication analysis tools. Systemic Elucidation and Assessment of Regulatory Cell-to-cell Interaction Networks (SEARCHIN) is an approach created to identify interactions between multicellular models of disease (Mishra et al., 2020). Mishra et al. identified in ALS models of neuron-astrocyte, a deleterious ligand-receptor pair, amyloid precursor protein (APP), and death receptor-6 (DR6). Therefore, multi-modal integrative bioinformatics approaches like SEARCHIN allow for the study of non-cell-autonomous mechanisms.

Central to this review is the use of iPSC-ALS models for drug discovery. Drugs under investigation, such as the acetyltransferase inhibitor, anacardic acid, was discovered through iPSC-ALS models (Egawa et al., 2012). ropinirole was identified through multi-phenotypic screening with high-content imaging using non-SOD1 fALS models from iPSCs (Fujimori et al., 2018). The combination of iPSC-ALS models and high-throughput drug screening with computational tools accelerates the development of ALS drugs (Lee et al., 2018). Neural networks (Congzhou et al., 2021) can be applied to predict small molecule targets to proteins of interest such as TDP-43, SOD1, and FUS. Drift is another tool from the Dokholyan lab that predicts protein targets of chemical compounds. In drug discovery, high-throughput screening methods are used to select potential candidates, that are computationally assessed though molecular modeling. Moreover, in drug design, the conformational changes induced by ligand binding are often difficult to capture and calculating the configurational entropy may pose a challenge. To circumvent these limitations, flexible docking approaches such as MedusaDock (Yin et al., 2010; Wang and Dokholyan, 2019) allow modeling both ligand and receptor flexibilities. MedusaDock is well-suited for iPSC-ALS model studies as it conformationally samples small molecules. Finally, Erebus (Shirvanyants et al., 2011) validates the binding site of the protein of interest. With the power of computation coupled with better disease-reproducible models such as iPSCs cultured in 3D-systems, the validity of the data is strengthened.

Regarding the clinical translation of bioinformatics, analysis of datasets investigating immune cells and gene alterations provided potential biomarkers for the much needed monitoring of ALS disease and treatment response (Xie et al., 2021). Investigations for unique ALS gene signatures compared to non-ALS controls led researchers to predict disease occurrence based on the combination of neurofilament-light (Nf-L) and neuroinflammatory biomarkers in the serum and cerebral spinal fluid these patients (Brodovitch et al., 2021). To date, the most accurate biomarker for ALS is the unique combination of Nf-L and neuroinflammatory markers in the serum and cerebral spinal fluid. Nf-L and cytokines possess long-term stability providing prognostic value as biomarkers for patients with neurogenerative symptoms (Huang et al., 2020). Also, the development of genetic bioinformatic tools affect clinical decisions such as genetic counseling and disease-risk assessment (Al-Chalabi et al., 2017). As stated, ALS presents a late-onset, however the disease may manifest much earlier, thus the discovery of specific and stage-sensitive biomarkers can aid the timely identification of the disease to efficiently intervene early symptoms and monitor the pathologies’ progression.

**FIGURE 5:**
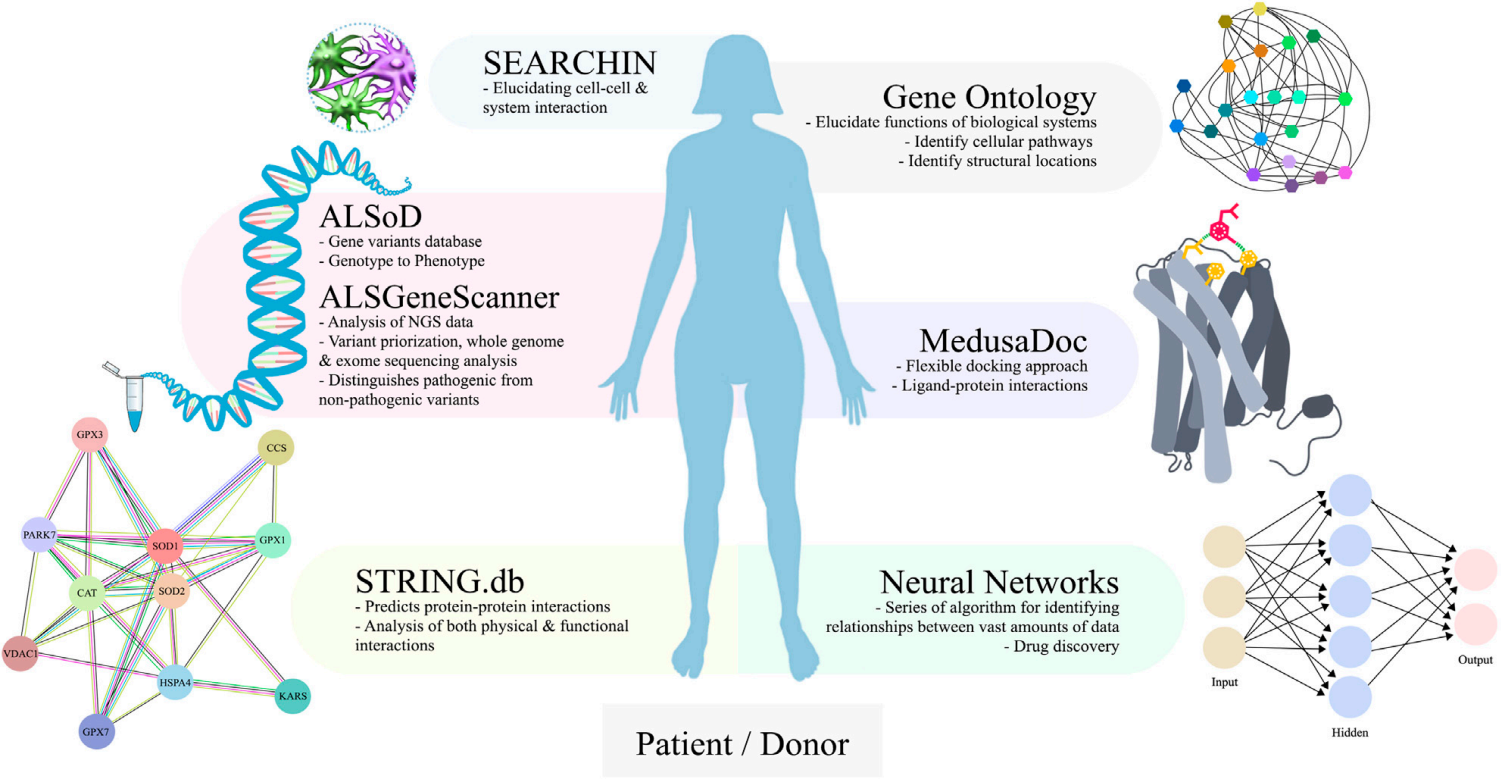
An overview of bioinformatics and computational tools that can be paired to iPSC-ALS research. For ALS genetics, bioinformatics repositories provide automated analysis for genotype to phenotype association studies (ALSoD) and interpretation of complex genetics data (ALSGeneScanner). For iPSC-ALS, protein-protein interactions can be mapped with subsequent pathway analysis (STRING.db). Scaling up to multicellular systems requires increased analysis techniques that are available (SEARCHIN). For iPSC-ALS drug discovery, small molecule targets to proteins of interest can be identified (Neural Networks) and docked with the ligand (MedusaDock).

ALS is a complex disease without a clear understanding of targetable disease-curable pathways. Therefore, it is imperative to highlight the critical axis for ALS pathogenesis. The application of bioinformatics and computational approaches such as ALSoD, ALSGeneScanner, multi-omics analysis, STRING.db, SEARCHIN, neural networks, DRIFT, and MedusaDock pushes the Frontier of our understanding of ALS. SEARCHIN applied to iPSC co-cultures of ALS patients elucidated our understanding of cell-cell interactions, similarly STRING.db permits the use of proteins extracted from iPSC-ALS to understand specific protein-protein interactions. Neural networks, DRIFT, Erebus, and MedusaDock allow for identification of new drugs, prediction of drug targets to proteins of interest, binding site validation, and docking and modeling of proteins or small molecules with iPSC-ALS cultures for *in vivo* validation experiments (Figure 5). Bioinformatic and computational techniques paired with iPSC-ALS experiments empowers streamlining drug discovery of iPSC-ALS cultures.

## Conclusion

Since their discovery, iPSCs’ public interest has centered on their use as therapeutic agents. However, unlike their multipotent counterparts, the mesenchymal stem cells, iPSCs’ clinical translation is still in preliminary stages. The lagging in the implementation of iPSCs in a clinical setting is due to the discussed limitations that include genetic instability, lack of standardized production, quality control, and robust xeno-free clinical-grade protocols. We believe that iPSCs’ true impact resides in their use as disease models and drug screening platform for pharmaceutical development. In addition to their clear advantage to model the donors’ genetic and epigenetic identity, iPSCs allow for scaling-up and high-throughput drug screening processes. Perhaps one of their most exciting features, is their capability to bypass animal experimentation in drug trials, which translates to a substantial reduction of the time, cost and ethical concerns associated with animal use. However, for this system to be widely implemented in the drug discovery field, iPSCs culture standardization demands must be rigorously met. Defined differentiation protocols, along with stringent characterization and use of isogenic controls can significantly assist in the systems reliability.

While continuous improvement of iPSC production and a deeper understanding of the cell differentiation process allows us to search for shared protocol standards and define criteria, a new set of tools involving 3D-culture and bioinformatics are emerging. Using 3D-cultures we can increase the resemblance of the in vivo physiological microenvironments, and thus, greatly enhance our understanding of the diseases’ biology and drug response. While bioinformatics can aid us in unveiling the diseases’ pathological mechanisms and in predicting new therapeutic compounds and their combinations for effective treatments. The incorporation of both these tools have the potential to shift the current limitations of ALS research and spark the discovery of new drugs and treatments. However, despite these clear advantages, 3D-modeling and bioinformatics integration to iPSCs-ALS research is rarely found. From scarce postmortem samples to in bulk-patient specific iPSCs that differentiate into any cell type of the brain, in vitro technology and their analysis tools have moved forward with giant leaps. We are now in need of the next step, towards the third dimension and integrated in silico analysis.

## Author’s notes

Illustrations were created using Adobe Photoshop (2020), and chemical molecules were modeled with PubChem Sketcher V2.4 (2021) by MA.
